# Assessment of acute pain and its management in an urban emergency department in Ghana

**DOI:** 10.1371/journal.pone.0343797

**Published:** 2026-03-20

**Authors:** Kwabena Antwi-Donkor, Jonathan Boakye-Yiadom, Godfred Boakye, Ama Antwi-Donkor, Abigail Mensah Hammond, Johnpaul Amenu, Enoch Opoku Afriyie, Richard Delali Agbeko Djochie, Michael Arthur Ofori, Ronald Feldman. Maio

**Affiliations:** 1 Accident and Emergency, Goldfields Mines Hospital, Tarkwa, Ghana; 2 Department of Epidemiology and Biostatistics, Kwame Nkrumah University of Science and Technology, Kumasi, Ghana; 3 Ghana Military Academy, Ghana Armed Forces, Accra, Ghana; 4 Directorate of Anaesthesia and Intensive Care; Komfo Anokye Teaching Hospital, Kumasi,; 5 Department Accident and Emergency, Komfo Anokye Teaching Hospital, Kumasi, Ghana; 6 School of Public Health, Johns Hopkins Bloomberg, Baltimore, Maryland, United States of America; 7 Department Pharmacotherapeutics and Pharmacy Practice, School of Pharmacy and Pharmaceutical Sciences, University of Cape Coast, Cape Coast, Ghana; 8 School of Public Health, University of Memphis, Memphis, Tennessee, United States of America; 9 Department of Emergency Medicine, University of Michigan Medical School, Ann Arbor, Michigan, United States of America; Stanford University School of Medicine, UNITED STATES OF AMERICA

## Abstract

**Introduction:**

Acute pain is classified as pain that lasts less than three to six months. Globally, pain is the third most common health problem with more than a quarter of patients reporting to the Emergency Department (ED) with pain-related chief complaints. This study aimed to determine the prevalence of acute pain and assess the pain management practices in the Komfo Anokye Teaching Hospital Emergency Department (KATH ED).

**Methods:**

Using the Numeric Rating Scale (NRS), the characteristics of acute pain among 378 patients presenting to KATH ED were measured. Additionally, the waiting time for the first pain treatment was calculated for each patient. Pain scores (pre- and post-treatment) were also taken to further inform patients’ satisfaction.

**Results:**

Out of 378 patients, 76% [95% CI: 71.3–80.2] reported to the ED with severe pain, 21% [95% CI: 16.9–25.4] reported with moderate pain, and 3% [95% CI: 1.5–5.1] reported with mild pain. The average waiting time for the initial assessment of pain was 83.97 minutes while the average waiting time for the administration of analgesia was 184.07 minutes. Having completed primary (AOR, 5.36; 95% CI, 1.03–27.97), JHS (AOR, 5.8; 95% CI, 1.19–28.35), SHS (AOR, 7.24; 95% CI, 1.38–38.01) and tertiary (AOR, 9.42; 95% CI, 1.60–55.62) were predictive of Door‑to‑Analgesia (DTA) time ≤ 90 minutes. Nearly three quarters of the study participants had maximum satisfaction with the pain management services in the ED.

**Conclusion:**

The study revealed that documentation of pain severity scores of patients presenting with acute pain at KATH ED was encouraging, however, most patients did not receive timely pain relief. The average waiting time for the initial assessment of pain as well as the administration of analgesia was extremely prolonged. Despite this, three out of every four of the study participants had maximum satisfaction for the overall pain treatment services in the ED. These findings suggest that pain management practices at KATH ED need improvement.

## Introduction

Pain is one of the most common complaints among patients presenting to Emergency Departments (EDs) with a global prevalence rate of 78% [1–3]. Also, according to [3], Pain is the primary reason for nearly four out of five (78%) emergency department visits. The presentation of acute pain is a generally recognised problem in ED practice, with some studies estimating that up to 70% of ED patients may present with pain [1,4]. Globally, pain is the third most common health complaint, with over three-quarters of patients presenting to the ED with pain-related chief complaints. An increasing burden of pain falls on Low- and Middle-Income Countries (LMICs) who are comparatively less equipped to provide adequate pain management [5]. In the year 2001, as part of global efforts to tackle the widespread challenges in pain management, the Joint Commission on Accreditation of Healthcare Organisations (JCAHO) introduced standards for healthcare organisations regarding the evaluation and treatment of pain [6].

Unrelieved pain could result in multiple adverse effects, both somatic and psychological. For example, the severe pain of fractured ribs results in inadequate respiratory effort, predisposes to atelectasis, and may cause hypostatic pneumonia leading to life-threatening hypoxia [7]. Patients with unrelieved pain sleep poorly, remain anxious, cooperate poorly with the treatment regimen, may be dissatisfied with care, and may even manifest signs of aggression towards healthcare staff [8]. These adverse effects of unrelieved pain militate against effective healthcare provision and prolong the length of stay in health facilities with attendant increases in cost for patients and hospitals [8]. For these and other reasons, acute pain and its associated management has been rightly recognised as a public health problem and as a human rights issue [9].

Most studies in the literature on acute pain management are reported from the USA, Europe, and Australia which are high-income countries (HICs) [1,4,10–13]. Studies on pain in LMICs like Ghana are largely palliative care oriented, however acute pain management and its timeliness have received little attention coupled with the challenge of overcrowding at the ED [5]. To address this gap, this study aimed to assess the prevalence of acute pain and the pain management practice in a tertiary health facility in Ghana.

Given the findings from several studies concerning poor acute pain management, a thorough assessment of pain management practice may help identify the specific parameters or areas in the treatment of pain that could be improved, to facilitate more effective and efficient pain care in the ED.

## Materials and methods

### Setting

This research was conducted in Kumasi in the Ashanti Region of Ghana. The study was conducted specifically at Komfo Anokye Teaching Hospital (KATH). Komfo Anokye Teaching Hospital (KATH) is a 1,200-bed tertiary service delivery point in Kumasi. KATH provides health care for about a third of Ghana’s population, making it the second- largest- hospital in the country. It serves as the main referring hospital for the Ashanti Region, Bono East, Ahafo, and Bono region, and sometimes the Northern Regions (Upper East, Upper West and Northern Regions) [14]. KATH is affiliated with Kwame Nkrumah University of Science and Technology (KNUST), which is also located in Kumasi. The Emergency Department at KATH was established in 2008 and provides 24-hour emergency services (surgical, trauma and medical emergencies). The ED has been divided into three distinct zones (Red, Orange and Yellow) based on the South African Triage Scale (SATS) [15]. The ED at KATH is well-equipped when compared with other district or regional-level facilities in Ghana.

### Inclusion/Exclusion criteria

The study included all patients who visited the KATH ED throughout the study period between April 2018 to September 2018, had acute pain, consciousness, ages 18–65, good mental health and consented to participate in the study. Patients who were referred to from other medical facilities were excluded.

### Sampling

A systematic sampling approach was adopted in selecting the patients. The recruitment of the study participants was done at triage. During the triaging of patients, respondents with acute pain as their cause for ED attendance were identified by the research assistants. Every third person who visited the ED with acute pain and met the inclusion criteria was selected after the person had consented to take part in the study.

**Fig 1:**
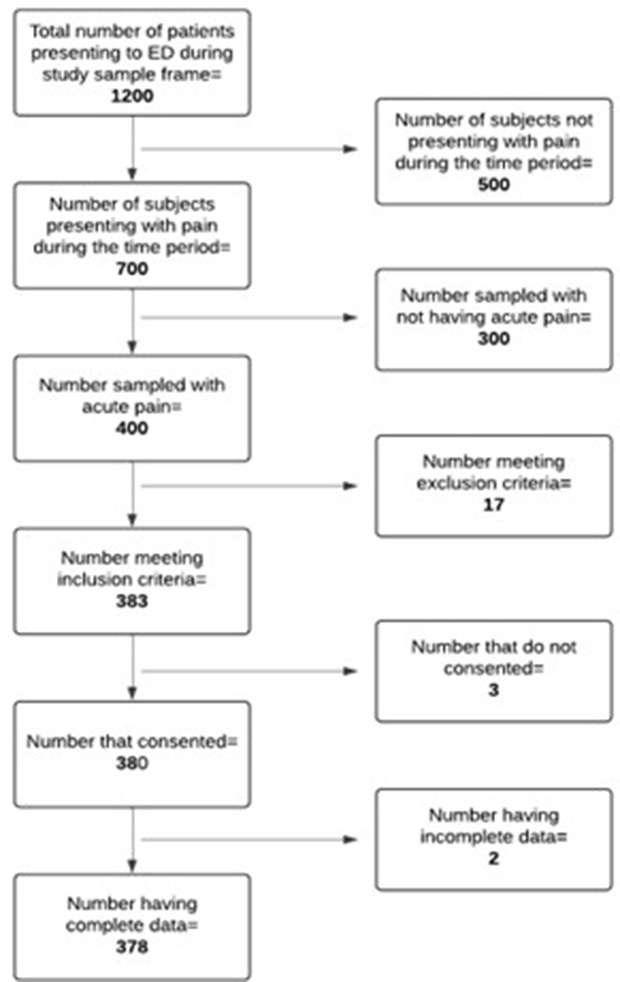
Flow chart of patient recruitment.

This selection method was followed until all the number of respondents required were obtained as shown in Fig 1. When a patient however fell in the interval but decided not to take part in the study, the individual was dropped. The next person presenting after the dropped individual was selected as a replacement, and the sequence was repeated with the same interval.

### Assessment tool and data collection

Data on the pain management practices were captured using a structured questionnaire. Data were recorded from the respondents electronically using electronic tablets and smartphones. Information about the respondent’s socio-demographic characteristics (age, sex, education, and occupation) and pain characteristics were recorded before analgesics were administered. To assist respondents in accurately identifying the anatomical location of their pain, a pictorial diagram was employed. Body parts on the diagram were numerically labeled to minimize data entry errors and ensure consistency in reporting. The research assistants administered the initial pain assessment tool during the triaging of the respondents. Patient satisfaction was solely based on the patient’s discretion; the choice of satisfaction level was under no circumstance influenced by research assistants. The survey tool was translated from English to spoken Twi and back- translated into English to ensure the validity of the verbal translation by each interviewer. Data collection commenced on April 11, 2018, for a period of 6 months. Data collection ended on September 30, 2018.

### Pain assessment

Pain intensity was measured using the Numerical Rating Scale (NRS), an 11-point scale ranging from 0, indicating no pain, to 10, representing the worst imaginable pain. This tool was chosen for its sensitivity and simplicity [16,17]. Patients verbally reported their pain levels during face-to-face interviews, reflecting the most severe pain experienced within the past 24 hours. Based on their responses, pain severity was categorized as mild (scores 1–3), moderate (scores 4–6), or severe (scores 7–10) [17,18].

### Pain management evaluation

Pain management interventions were monitored by recording the type and dosage of analgesics administered, although the study did not enforce adherence to any specific clinical protocols. The effectiveness of the administered analgesics was evaluated by reassessing pain intensity at 30-minute intervals for a total duration of two hours post-treatment.

Additionally, patient satisfaction with pain management was assessed using a 4-point Likert scale, ranging from 1 (no satisfaction) to 4 (maximum satisfaction). This measure was entirely based on the patient’s subjective evaluation, without any influence from the researchers.

### Clinical context

Although the study did not explicitly evaluate compliance with established clinical guidelines such as the WHO analgesic ladder, it assessed pain management using a combination of objective, subjective, and process measures. Objective evaluation focused on the reduction in NRS scores following treatment. Subjective assessment was based on patient-reported satisfaction, while process evaluation examined the frequency of pain reassessment in line with recommended best practices for monitoring acute pain.

### Covariates

The study accounted for potential confounding variables by adjusting for both sociodemographic and clinical factors. Sociodemographic variables included gender, age, religion, marital status, occupation, and ethnicity. Clinical variables included triage zone, presenting complaints, pain score, and the location and duration of pain.

### Recruitment and training of research assistants

The study employed four research assistants to help the principal investigator in administering the questionnaires. These assistants were recruited based on the following eligibility criteria: have completed a first degree, are proficient in both English and Twi language, competent user of a smartphone or an electronic tablet computer. Other qualities, such as the ability to demonstrate a high sense of maturity and self-confidence, were also considered. They were given two days of training on the tools based on the NRS guidelines. During the training, the research assistants were given time to familiarize themselves with the electronic way of capturing data. The research assistants were also trained to ask questions about the right way to prevent bias due to instrumentation. A mock interview was organized among the team to agree on the common words to use during the data collection period, especially when the need arose to translate from English to Twi. After pre-testing the questionnaire, another meeting was organized to effect the changes and resolve the problematic and ambiguous questions encountered during the pre-testing phase.

### Pain treatment

Pain treatment modalities in the emergency department included both narcotic and non-narcotic options, administered through various routes. Narcotic options included intravenous and subcutaneous injections of medications such as morphine, as well as oral administration of narcotics like codeine. Non-narcotic treatments included oral medications such as ibuprofen and acetaminophen. The treatment choice was guided by the clinical judgment of the treating physician, considering the severity of pain, the patient’s medical history, potential side effects, and the need for rapid pain relief.

In the ED, narcotic options for pain management include intravenous and subcutaneous injections of medications such as morphine as well as oral administration of narcotics like codeine. Doses of Morphine administered are 2–10 mg IV every 2–6 hours as needed. The dosing for subcutaneous is similar to that of the morphine.

It is highly effective for relieving moderate to severe pain and acts rapidly, especially when administered intravenously. Potential side effects of the morphine can include nausea, vomiting, constipation, respiratory depression, sedation, dizziness and a risk of dependency or abuse. For Codeine, is often used for mild to moderate pain relief at doses of 15–60 mg orally every 4–6 hours, with a maximum daily dose of 350 mg. While it is effective, it has a slower onset compared to morphine and shares similar side effects, including constipation, drowsiness, nausea, vomiting and potential dependency with prolonged use.

Non-narcotic treatment options include oral medications such as ibuprofen and acetaminophen. Ibuprofen is commonly prescribed at doses of 200–800 mg every 6–8 hours, with a maximum daily dose of 3200 mg. It is particularly effective for mild to moderate pain and inflammation and can reduce fever if present. Side effects that may limit its use in certain patients include gastric irritation, dyspepsia, gastrointestinal ulcers, and an increased risk of gastrointestinal bleeding. In addition, Ibuprofen can impair kidney function in susceptible individuals. Acetaminophen is another non-narcotic option, usually given at doses 325–1000 mg every 4–5 hours, with maximum daily dose of 4000 mg (or 3000 mg/day for chronic use). It is effective for mild and moderate pain and fever reduction, and it is safer alternative for patients with gastrointestinal sensitivities.

However, excessive or prolonged use can lead to hepatotoxicity or liver damage. For severe pain, morphine is often preferred because it has a rapid and potent effect. For mild to moderate pain, ibuprofen or acetaminophen may suffice and are associated with fewer risks of dependency. The choice of treatment depends on patients -specific factors such as medical history, contraindications and clinical judgement of the treating physician. In some cases, combination therapy is employed, where non-narcotics such as acetaminophen are combined with narcotics like codeine. This approach can enhance pain relief while reducing the required dose of the narcotic, thereby, minimizing dependency risks. Physicians considered both pharmacological efficacy and patient safety in making these decisions, ensuring that pain management was tailored to the specific clinical needs of each patient.

### Data analysis and management

Data were collected electronically and exported to Microsoft Excel v16 (Microsoft Corporation, USA) for initial cleaning and formatting. The cleaned dataset was then imported into Stata v14 (StataCorp, USA) for statistical analysis. During data processing, variables were checked for consistency, completeness, and outliers. Descriptive summaries and frequency checks were conducted to identify missing values or erroneous entries. Cases with missing key outcome variables were excluded from the final analysis, while missing values in covariates were addressed using listwise deletion, assuming data were missing at random (MAR).

Door to Analgesia (DTA) time was categorized into two groups: less than or equal to (≤) 90 minutes, and more than (>) 90 minutes. While international guidelines (NHMRC, BAEM) recommend DTA times of 30 minutes or less for patients with severe pain, we adopted the 90-minute threshold based on the median DTA reported in a prior U.S. multicentre study, recognizing that even this extended timeframe represents a pragmatic, though still suboptimal, benchmark for LMIC settings. Importantly, this 90-minute threshold is three times longer than recommended guidelines and should not be interpreted as an acceptable standard of care.. Bivariate analyses (e.g., chi-square tests, t-tests) were initially conducted to examine associations between predictor variables and the outcomes of interest, followed by multivariate logistic regression models to identify independent predictors of timely analgesia (DTA ≤ 90 minutes) and patients’ maximum satisfaction with care. Assumptions for statistical analyses, including linearity in the logit for logistic regression, independence of observations, and absence of multicollinearity among predictors, were tested and met. Variables were retained in multivariate models based on theoretical relevance and statistical significance in bivariate analysis.

All hypothesis testing was two-tailed, and statistical significance was determined at a p-value of less than 0.05 with 95% confidence intervals reported for all estimates to reflect the precision of associations.

### Sample size

For this study, the prevalence of pain was adopted from a similar study conducted in the USA, which indicated a prevalence of 78%. The estimation of the sample size was based on the Bland formula for calculating a sample for a single proportion as shown below: n = Z²p(1-p)/d² where:

n = sample size

Z = 95% confidence interval

P = proportion

d = margin of error

The sample size for the present study was calculated as;

n = Z^2^p(1-p)/d^2^ = N = (1.96)^2^ * (0.78)(0.22)/0.05^2^ = 0.65921856 = 263.68

Where

n = the desired sample size

P = 0.78 assumed the proportion of patients presenting with pain at the ED

d = 0.05 desired precision

Non-response rate = 20%

A sample size of 264 was estimated, and with a presumed 20% non-response rate, a sample size of 317 was estimated for the present study.

### Ethics

Approval for the study was provided by the ethical committee of the Kwame Nkrumah University of Science and Technology with reference number (CHRPE/AP/102/18). Permission was equally obtained from the Komfo Anokye Teaching hospital prior to data collection. Participants were then introduced to the project. Patients who understood and agreed to be part of the study signed a written consent form.

## Results and discussion

Three hundred and seventy-eight subjects presented with acute pain constituting a prevalence of 33.3% [400/1200), 95% CI: 30.66–36.08]. The male to female was approximately 3:1, with an average age of 36.5 years (SD = 12.18). The largest occupational group among participants was traders (28%), followed by artisans (22%), and 10% were professionals in various fields. In terms of education, 45% of the patients had completed Junior High School. The marital status of the participants revealed that 51% were married, 2% were widowed, 4% were cohabiting, and 43% were single. Most participants (83%) identified as Christians, 14% were Muslim, and 3% had no religious affiliation.

Regarding their medical conditions, 30% (114 patients) presented to the emergency department with fractures, 27% with abdominal pain, and 20% with wounds. Trauma cases accounted for 65% of admissions. The majority of patients (78.84%) were triaged into the yellow zone, and over half of the participants had not subscribed to the National Health Insurance (Table 1).

**Table 1:** Demographic and characteristics of patients presenting to KATH with pain, Ghana (N = 378).

Variable	Frequency (N = 378)	Percentage (%)
Age, mean (SD) years	36.5 (SD = 12.18)	
Age group		
18–27 years	97	25.66
28–37 years	120	31.75
38–47 years	87	23.02
48–57 years	47	12.43
58–65 years	27	7.14
Gender		
Male	278	73.54
Female	100	26.46
Educational status		
None	29	7.67
Primary	54	14.28
JHS	169	44.71
SHS	68	17.99
Vocational	8	2.12
Tertiary	50	13.23
Marital status		
Single	161	42.59
Married	194	51.32
Cohabiting	15	3.97
Widow/Widower	8	2.12
Occupational status		
	21	5.56
Artisan	85	22.49
Farmer	43	11.38
Professional	37	9.79
Retired/Pensioner	3	0.79
Service/Sales worker	59	15.61
Student	23	6.08
Trader	104	27.51
Other	3	0.79
Religion		
None	10	2.65
Christian	314	83.07
Muslim	53	14.02
Traditional	1	0.26
NHIS subscription		
Yes	181	47.88
No	197	52.12
Cause of Admission		
Trauma	247	65.34
Non-Trauma	131	34.66
Triage zone		
Yellow	298	78.84
Orange	79	20.90
Red	1	0.26
Causes of Pain		
Wounds/Lacerations	78	20.63
Fractures	114	30.16
Dislocation	13	3.44
Burns	2	0.53
Sprain	17	4.50
Blunt abdominal pain	23	6.08
Non-traumatic abdominal pain	80	21.16
Others*	51	13.50

* Cellulitis, abscesses, haemorrhoids, and testicular torsion

### Severity of pain

There was a notable reduction in pain among patients after the administration of analgesia. The average pain score before receiving analgesia was 7.83 (SD ± 1.95), which decreased to 3.89 (SD ± 1.81) after treatment. This represents approximately a 50% reduction in pain severity as shown in Table 2.

**Table 2:** Pain Severity using NRS among Patients at KATH ED.

Variable	Pain Score at ED Presentation	Post Analgesia Pain Score
	Mean (± SD)	Mean (±SD)
N = 378	7.83 (SD ± 1.95)	3.89 (SD ± 1.81)
Gender		
Male	7.88 (1.96)	3.76 (1.81)
Female	7.70 (1.92)	4.28 (1.75)
Age		
18-27	7.93 (1.91)	3.71 (1.55)
28-37	7.90 (1.92)	3.89 (1.86)
38-47	7.89 (1.98)	4.14 (1.89)
48-57	7.43 (2.01)	3.60 (1.97)
58-65	7.74 (2.05)	4.30 (1.88)
Triage zone		
Yellow	7.57 (1.91)	3.66 (1.73)
Orange	8.78 (1.78)	4.76 (1.88)
Red	10.00	5.00

### Timeliness of analgesia administration

According to Table 3, twenty-two female patients (22%) had a DTA time of ≤ 90 minutes. After adjusting for predefined covariates, females were 11% more likely to have a DTA time of ≤ 90 minutes, though this finding was not statistically significant (AOR, 1.11; 95% CI, 0.55–2.22).

**Table 3:** Predictors of DTA time ≤ 90 minutes – bivariate and multivariate analyses.

Variable	DTA time ≤ 90 mins (%)	OR	95% CI	AOR	95% CI
Gender					
Male	77(22.70)	Ref		Ref	
Female	22(22.00)	0.74	0.43-1.26	1.11	0.55-2.22
Age					
18-27	31 (31.9)	Ref			
28-37	31(25.83)	0.74	0.41-1.34	0.66	0.30-1.42
38-47	22(25.29)	0.72	0.38-1.37	0.88	0.36-2.19
48-57	7(14.89)	0.37	0.15-0.92*	0.43	0.13-1.43
58-65	8 (29.63)	0.9	0.35-2.27	0.99	0.26-3.72
Educational status					
None	2(6.90)	Ref		Ref	
Primary	13(24.07)	4.28	0.89-20.49	5.36	1.03-27.97*
JHS	44(26.04)	4.75	1.09-20.81*	5.8	1.19-28.35*
SHS	21(30.88)	6.03	1.31-27.74*	7.24	1.38-38.01*
Vocational	2(25.00)	4.5	0.52-38.65	5.73	0.57-57.80
Tertiary	17(34.00)	6.95	1.47-32.8*	9.42	1.60-55.62*
Occupation					
Unemployed	5(23.81)	Ref		Ref	
Farmer	18(21.18)	0.85	0.24-2.94	0.55	0.13-2.35
Professional	9(20.93)	1.6	0.12-21.59	0.65	0.04-11.06
Sales worker	12(32.43)	1.54	0.45-5.19	0.82	0.19-3.58
Trader	1(33.33)	1.6	0.12-21.56	1.34	0.05-34.83
Artisan	22(37.29)	1.9	0.61-5.92	1.25	0.33-4.68
Student	5(21.74)	0.9	0.22-3.64	0.29	0.06-1.50
Retired	26(25.00)	1.07	0.36-3.20	0.67	0.19-2.37
Other	1(33.33)	0.86	0.28-2.66	0.54	0.15-1.99
NHIS subscription					
Yes	42(23.20)	Ref		Ref	
No	57(28.93)	1.35	0.84-2.14	1.37	0.79-2.38
Triage Zone					
Yellow	65(21.81)	Ref		Ref	
Orange	33(41.77)	2.57	1.52-4.35*	2.35	1.23-4.50*
Red	1(100.00)	1		1	
Causes of ED Admission					
Abdominal pain	20(19.42)	Ref		Ref	
Fracture	31(27.19)	1.91	0.92-3.96	1.51	0.66-3.45
Burns	0(0.00)	1		1	
Dislocation	5(38.46)	3.62	1-13.07	1.44	0.33-6.29
Sprain	5(29.41)	1		1	
Wound/ Laceration	26(33.33)	2.98	1.13-7.86*	3.82	1.24-11.77*
Other^	12(26.19)	1.94	0.92-4.11	1.7	0.75-3.84
Pain severity before Analgesia					
Mild	1(10.00)	Ref		Ref	
Moderate	13(16.46)	1.77	0.21-15.22	2.46	0.24-25.13
Severe	85(29.41)	3.72	0.47-30.06	4.88	0.50-47.23

* Statistically significant at ⍺= 0.05

Educational attainment was predictive of shorter DTA times, with those having completed primary (AOR, 5.36; 95% CI, 1.03–27.97), Junior High School (AOR, 5.8; 95% CI, 1.19–28.35), Senior High School (AOR, 7.24; 95% CI, 1.38–38.01), and tertiary education (AOR, 9.42; 95% CI, 1.60–55.62) more likely to have DTA times of ≤ 90 minutes compared to those with no formal education. Patients triaged to the orange ward were twice as likely to have a DTA time of ≤ 90 minutes (AOR, 2.35; 95% CI, 1.23–4.50). Presenting to the ED with wound or laceration-related pain was also predictive of a DTA time of ≤ 90 minutes in both bivariate and multivariable models (AOR, 3.82; 95% CI, 1.24–11.77). There was no significant evidence for other covariates, including pain severity before analgesia and occupation.

In summary, only 26% (99/378) of all patients received analgesia within 90 minutes of ED arrival, meaning that 74% of patients experienced delays exceeding even this extended benchmark, which itself is three times longer than the 30-minute standard recommended by NHMRC and BAEM guidelines. These findings highlight significant gaps in timely pain management at the study site.

### Pain management satisfaction

The multivariate analysis (Table 4) revealed that females (AOR, 0.51; 95% CI, 0.29–0.88), those not subscribed to the NHIS (AOR, 0.90; 95% CI, 0.53–1.52), and patients triaged to the orange zone (AOR, 0.68; 95% CI, 0.34–1.36) had reduced odds of achieving maximum satisfaction. However, these findings were not statistically significant. Being a student was strongly predictive of maximum satisfaction in the bivariate model, but after adjusting for predefined covariates, it was no longer statistically significant (AOR, 10.31; 95% CI, 0.30–2.86).

**Table 4:** Predictors maximum satisfaction.

Variable	OR	95% CI	AOR	95% CI
Gender				
Male	Referent		Referent	
Female	0.65	0.40- 1.07	0.72	0.38-1.35
Age				
18-27	Referent			
28-37	0.63	0.34-1.17	0.67	0.31-1.43
38-47	0.73	0.37-1.41	0.68	0.28- 1.65
48-57	0.69	0.32-1.52	0.67	0.22-2.00
58-65	1.29	0.44-3.80	0.93	0.24-3.66
Educational status				
None	Referent		Referent	
Primary	1.25	0.48- 3.27	0.87	0.30-2.57
JHS	1.25	0.54- 2.88	0.67	0.25-1.80
SHS	1.71	0.66- 4.42	0.71	0.23-2.16
Vocational	1.58	0.27-9.31	1.10	0.17-7.27
Tertiary	2.11	0.75- 5.90	0.89	0.22-4.00
Occupation				
Unemployed	Referent		Referent	
Farmer	1.89	0.59-6.07	1.80	0.48-6.74
Professional	1.56	0.48-5.05	1.29	0.30-5.46
Sales worker	1.24	0.42-3.59	1.06	0.32-3.50
Trader	1.02	0.38-2.79	1.00	0.34-2.94
Artisan	1.07	0.39- 3.00	0.93	0.33-4.68
Student	11.00	1.22-99.26*	10.31	0.30-2.86
Retired	1.00		1.00	
Other	1.00		1.00	
NHIS subscription				
Yes	Referent		Referent	
No	0.86	0.55-1.36	0.90	0.53-1.52
Triage Zone				
Yellow	Referent		Referent	
Orange	1.16	0.66-2.04	0.68	0.34-1.36
Red	1.00		1.00	
Causes of ED admission				
Abdominal pain	Referent		Referent	
Wounds/Lacerations	1.06	0.55-2.05	1.16	0.56-2.42
Fracture	0.92	0.51-1.65	1.03	0.53-1.99
Burns	1.00		1.00	
Dislocation	2.15	0.45-10.32	2.38	0.43-13.12
Sprain	1.27	0.38-4.22	1.45	0.37-5.68
Other	1.03	0.49-2.19	0.93	0.40-2.12
Waiting time before analgesia				
> 90 minutes	Referent		Referent	
≤ 90 minutes	1.26	0.74- 2.13	1.22	0.67-2.22

Patients presenting with dislocations (AOR, 2.38; 95% CI, 0.43–13.12), sprains (AOR, 1.45; 95% CI, 0.37–5.68), and a DTA time of ≤ 90 minutes (AOR, 1.22; 95% CI, 0.67–2.22) showed higher odds of satisfaction, although these associations were not statistically significant.

## Discussion

This study is the first comprehensive analysis of Emergency Department pain in Ghana as well as low/middle-income countries in Sub-Sahara Africa. The results demonstrate a marked decline in the severity of pain after patients received analgesics. Additionally, majority of the patients received analgesics in more than 90 minutes after arriving at the ED and patients who received analgesics in less than 90 minutes expressed maximum satisfaction to the pain management. Study has reported that 30% to 64% of patients undergoing back surgery have experienced moderate to severe pain on the first postoperative day [19]. This aligns with findings that acute postoperative pain scores can average around 5 on a numerical rating scale (NRS), indicating a considerable incidence of moderate pain in surgical cohorts [20]. Furthermore, the complexity of surgical procedures is often correlated with increased pain severity, underscoring the need for tailored analgesic protocols post-operation [21–22].

The gender composition of patients presenting to KATH ED with acute pain revealed that more males, reported with acute pain as compared with females. Similar findings have been reported in other studies conducted in LMICs like Burkina Faso, Ethiopia and Nigeria [23–25]. This contradicts other findings of studies conducted in HICs like Canada and USA especially the PEMI study [1,4].

Assessment and documentation of pain scores are requirements for effective pain management. Documentation should cover the initial as well as subsequent post analgesic re-assessment of pain [25]. For the present study, pain assessment or evaluation was done for all the patients at triage. However, in the study conducted by Tanabe et al. on ED pain management practices, pain assessment was not frequently done except for patients presenting with chest pain [1]. Similarly, in Kaboré’s study which involved assessment of acute pain in a trauma centre in Ouagadougou, only 44% of the patients had pain assessment [22].

In 2009, a systematic review identified several factors as causes of ineffective pain management in the ED. These factors included failure to acknowledge pain, non-assessment of initial pain, the lack of guidelines for pain management, non-documentation of pain and non-assessment of the adequacy of pain treatment [26]. The Australian National Health and Medical Research Council (NHMRC) with the view of standardising pain management and its improvement, recommended the documentation of pain scores and a median time to analgesia of 30 minutes for patients with acute pain presenting to the ED [10].

The British Association for Accident and Emergency Medicine (BAEM) guidelines for the management of acute pain recommend that patients with severe pain should receive appropriate analgesia within twenty minutes at the ED [27]. In the present study, the majority of patients experienced a reduction in their pain score by half or more after receiving analgesia. However, without detailed data on specific medications, doses, and adherence to established analgesic protocols such as the WHO pain ladder, we cannot definitively assess whether optimal pharmacological management was achieved. The inadequate pain relief may be attributed to the underdosing of analgesics. It is possible that the administered analgesics were not titrated to effect. Additionally, some patients may not have received analgesia appropriate to the severity of their pain. In some cases, the suboptimal outcome could also be due to the selection of an inappropriate route of administration.

According to the BAEM guidelines on pain management, patients with severe pain should receive adequate intravenous opiate preferably morphine or rectal NSAID supplemented by an oral analgesic [27]. The guidelines also advocate for an oral NSAID or codeine phosphate for patients with moderate pain [27]. The findings in this study indicate proper and frequent documentation of pain scores are a step in the right direction. This action facilitated health providers in making informed decisions on the right pain medication which resulted in the significant reduction in pain scores among patients. Similar practices should be extended to other health facilities across the country and in the sub-region.

The BAEM guidelines on acute pain state that all patients who complain of severe pain should receive intravenous narcotics within 20 minutes of presenting to the ED. The guidelines also advocate for oral analgesic at triage for patients with moderate pain [27]. The NHMRC guidelines on acute pain also recommend that patients in acute pain should receive documentation and treatment of pain with appropriate analgesia within 30 minutes of presenting to the ED [10]. The present study also revealed that the average time to initial administration of analgesia was 184.07 minutes (approximately 3 hours) for all patients, and 176.3 minutes (range: 10–710 minutes) for patients with severe pain.

Again, the study found that only 4% of patients with severe pain received pain medications within half an hour of presenting to the ED. Even when using the considerably relaxed 90-minute DTA threshold—three times the internationally recommended standard—the majority of patients in our study did not receive timely analgesia. This finding underscores the severity of delays in our setting and the urgent need for systematic intervention. Future quality improvement efforts should work progressively toward the evidence-based 30-minute standard, recognizing that this will require substantial system-level changes.

These findings are below the recommendation by the BAEM guidelines and that of the NHMRC on acute pain. Other studies also found DTA time below the BAEM and NHMRC acute pain management guidelines [27]. The multi-centre study from United States and Canada revealed a mean time to analgesia of 90 minutes (range 0–962 minutes) [4]. The average time to analgesia for the respondents in the Gondar University study was also found to be 61 minutes [23]. Even though the BAEM, NHMRC and American Pain Society (APS) guidelines have been indicated to be difficult to achieve in the ED, the current study’s average DTA of 184.07 indicate a very wide gap or disparity between LMIC EDs and that of the HICs in addition to the above-mentioned guidelines. Overcrowding of the ED and understaffing are often cited as the possible reasons for the delay in the evaluation and initiation of pain medications by physicians at the ED and this study is no different [28].

According to the South African Triage Scale (SATS), patients presenting with severe pain typically fall within the ‘orange’ or ‘red’ categories, for which treatment should be initiated within 10 and 0 minutes, respectively. However, the current study demonstrates a significant delay in the administration of analgesia, far exceeding these recommended timeframes. Empowering experienced emergency nurses to initiate analgesia at triage has been shown to significantly reduce time to pain management. Moreover, accurate triage using SATS helps ensure appropriate prioritization of care. In a prospective study conducted over a year, emergency nurses administered opioids to ED patients in acute pain [29].

The study concluded that the assessment and initiation of intravenous opioids by experienced emergency nurses could shorten the time patients wait to receive pain medications while in the ED. As LMICs continue to make strides on improving ED pain management, apart from making a conscious effort to expand EDs structurally and improving doctor- patient ratio at the EDs, efforts should be made to institute capacity building programs which help train ED nurses to at least start analgesics in order to reduce overcrowding. Additionally, further studies assessing the contributing factors of overcrowding and delay in DTA at the ED be conducted. The present study also found age and gender to be predictors of early DTA time. These variables were, however, not statistically significant at the bivariate level and multivariate level; however, they are worth discussing. Younger patients (27 years and below) were more likely than older patients (27–67 years) to have early DTA time. Younger patients were also more likely to have adequate pain relief when compared with older patients.

The study [30] by Jones et al., which assessed age as a risk factor for inadequate pain relief at the ED also concluded that elderly patients (mean 74 minutes) waited longer hours than their younger counterparts (mean 52 minutes) before receiving analgesics at the ED [27]. Also, females had 26% reduced odds of early DTA time when compared with males. This finding aligns with a study conducted by Chen et al. which assessed the gender difference in pain management in the ED. In Chen’s study, women waited much longer than men to receive analgesia with an average difference of 15 minutes even though they had similar pain scores [31]. This finding of this study however contradicts the findings of the prospective study by Raftery et al., which concluded that female patients reporting to the ED with acute pain are more likely to receive analgesics on time as well as receiving potent analgesics [32].

The disparity in the findings of the two studies could be attributed to the different pain threshold expressed by men and women across different variate regions. Even though both sexes may have the same pain scores, the one who manifests the pain more through physical expression is more likely to receive attention from a health provider. According to the present study, educational status and triage zone were also predictors for early DTA time at the multivariate level. A patient with educational level equal to or higher than primary school had a fivefold increased likelihood of having an early DTA time when compared with one with no formal education (AOR 5.36, 95% CI 1.03–27.97). The educational status of an individual has an influence on how an individual communicates and expresses himself. Educated patients thus, might have expressed their symptoms better resulting in earlier treatment or attention.

The triage zone was also another predictor for early DTA time. A patient triaged Orange had a twofold increased likelihood of having an early DTA time as compared with a patient triaged Yellow (AOR 2.35, 95% CI 1.23–4.50). Patients who triaged Orange need more urgent attention compared to those triaged Yellow. This finding conforms to the guidelines of the South African Triage Scale (SATS) which recommends clinicians to attend to patients in Orange early. Severe pain is also a discriminator to Orange in the SATS [15]. This current finding is in tune with the results of similar studies conducted in Africa. Amare et al., in a study conducted in Gondar University Hospital in Ethiopia, found that patients with severe pain received pain medications earlier than those with moderate and mild pain [18].

Rampanjato et al., in a study done in Central Africa, about the factors affecting pain treatment by caregivers also found that the severity of pain is highly linked with the average time to analgesia by nurses [33]. Results from this study reveal disproportionate demographic distribution in terms of timely pain management. Improving access to timely pain medication will require an urgent systematic and detailed explanation of barriers faced by individuals with a keen look at educational background and gender disparity.

Effective pain management is one of the factors that determine patient satisfaction with health care delivery. In the present study, nearly 75% of patients were fully satisfied with the entire process of pain management offered to them at the ED. Findings from the present study are consistent with the results of other studies. A survey by Harrel in an ED of Basse-Normandie, France revealed that 88% of the respondents were satisfied with the pain treatment given, even though only a tenth of them had their pain alleviated at discharge [34].

In the US, a study assessing the adequacy of pain relief pointed out a median patient satisfaction rating as “very good” [35]. However, the survey conducted in Ouagadougou found that a little over half of the patients enrolled in the study (58.6%) were satisfied with pain treatment received at the trauma centre. About a third (36.8%) of the respondents were not really satisfied, while 4.6% were totally dissatisfied with the management received [22]

Patient satisfaction with pain treatment received in the ED goes beyond just the administration of analgesia to reduce their pain. Patient satisfaction is not solely based on the pain treatment received but encompasses factors such as staff attitude and challenges that the patient encounters at the ED Thus, satisfaction could be positively influenced by respectful contact between patient and doctor. The perception that the nurse has time for the patient, listens to the patient’s concerns, and displays a compassionate caring attitude towards the patient and family may also have a positive impact on the patient’s satisfaction.

Again, from the present study, 58% of the respondents complained of residual moderate or severe pain after receiving medications, yet the majority of patients were satisfied with the overall pain treatment service they received. The support and compassion exhibited by a caregiver towards a patient reduces a patient’s anxiety and eventually leads to a lower pain rate. This support and kindness demonstrated by the health worker ultimately influence patients’ satisfaction [36].Based on this study, it can be understood that a combination of proper pain treatment (documentation of pain score, timely pain medication, effective pain medication) and positive staff attitude could have significantly increased the satisfaction levels of all patients. Further assessments might be helpful for pain management services at KATH and other LMIC hospitals to uncover and remedy problems that are affecting maximum pain management satisfaction at the ED.

A striking finding of this study is the discordance between objective pain management outcomes and patient satisfaction. Despite 58% of patients reporting residual moderate to severe pain after treatment and an average DTA time exceeding three hours, nearly three-quarters of patients expressed maximum satisfaction with their care. While staff compassion and attitude undoubtedly contribute to patient satisfaction, this paradox raises several critical concerns. First, high satisfaction scores may mask significant deficiencies in clinical outcomes, potentially creating false reassurance for healthcare administrators and reducing urgency for system improvements. Second, patients’ expectations may be shaped by prior experiences in resource-limited settings, leading to acceptance of suboptimal care as satisfactory. Third, cultural factors, power dynamics in the patient-provider relationship, or gratitude for any care received may influence satisfaction reporting independently of pain relief. Importantly, patient satisfaction should not be interpreted as evidence of adequate pain management. While we should continue to prioritize compassionate, respectful care, we must simultaneously address the objective failures in timely and effective pain relief. Quality improvement initiatives must focus on both dimensions—enhancing the clinical effectiveness of pain management while maintaining the positive interpersonal aspects of care that patients value.

Prior to drawing conclusions from this study, some limitations require consideration. Firstly, the present study was conducted in a single ED and may not represent the practice of all emergency centres. However, other studies from around the globe have shown similar results. Additionally, the goal of the study was not to conduct an exhaustive pain management assessment at several health facilities. Instead, we aimed using KATH which has one of the largest EDs in Ghana to identify strengths and weaknesses in pain management which needed future assessments. Secondly, the knowledge and attitudes of the patients and healthcare givers were not considered in the present study but have been shown in other studies to be factors that influence pain management in the ED.

We had to assume both patients and healthcare givers had a fair knowledge and attitudes in relation to pain management. Thirdly, the study did not capture information on doses of administered pain medication(s). Moreover, the study used 90 minutes judgement criteria for prolonged or short DTA based on the multicenter study conducted in US instead of NHMRC standard of 30 minutes. This was because the NHMRC and other standards like the APS have been seen to be difficult to achieve at the ED. Lastly, the study did not directly measure the contributing factors of overcrowding and delay in DTA. It is recommended that further studies be conducted on this subject matter. Despite limitations, these results provide a more useful assessment of pain management at the ED that can be used to improve health decision-making and facilitate effective and efficient pain care in LMICs like Ghana. For adequate pain management in the ED, there should be an in-depth understanding of the factors that affect the treatment of pain in ED patients. These factors or variables are often categorised into system and patient variables. System variables include the type of pain treatment (pharmacological and non-pharmacological), knowledge of ED personnel on pain management, attitudes of personnel towards pain management, regular training sessions for caregivers, availability of guidelines for pain management and other environmental factors (e.g., overcrowding in the ED) affecting the ED.

Patient variables are made up of physiological variables like intensity, location, and duration of pain. Patient variables also include demographic factors (age, gender and ethnicity) and psychological factors like mood and adherence or refusal to take medications [1].

### Limitations

Although this study offers valuable insights into acute pain management practices within a tertiary hospital in Ghana, several limitations must be acknowledged. First, the study did not evaluate adherence to established clinical guidelines such as the WHO analgesic ladder, nor did it capture detailed information on pharmacologic interventions, including specific drug classes and dosing regimens. Future studies should consider incorporating direct audits to assess guideline compliance more thoroughly. Second, the duration of pain reassessment was limited to two hours following analgesic administration. This short window may not adequately reflect longer-term outcomes or the need for additional or rescue analgesia. Third, patient satisfaction was measured through self-reported responses, which, while useful, may not directly correlate with objective measures of pain relief and could introduce response bias.

Additionally, the single-center design of the study may limit the generalizability of the findings to other healthcare settings, particularly those in non-tertiary or rural areas of Ghana. Finally, although the study adjusted for several key covariates, the influence of unmeasured confounding variables—such as prescriber preferences or the use of non-pharmacologic interventions—cannot be ruled out.

A significant limitation of this study is the absence of detailed pharmacological data, including specific drug classes, dosing regimens, frequency of administration, and titration practices. Additionally, we did not evaluate adherence to established clinical guidelines such as the WHO analgesic ladder. These gaps limit our ability to assess whether the observed pain relief was optimal or whether underdosing or inappropriate medication selection contributed to residual pain. Future studies should incorporate comprehensive medication audits and guideline adherence assessments.

## Conclusion

The present study revealed that a substantial number of patients presented to the KATH (ED) with severe acute pain and did not receive adequate or timely pain relief. Although most patients arrived at the ED with moderate to severe pain, more than half of the respondents reported experiencing residual moderate or severe pain even after receiving analgesia. Furthermore, only a few patients presenting with acute pain received analgesia within 90 minutes—a finding that contradicts the guidelines of the British Association for Emergency Medicine (BAEM), the National Health and Medical Research Council (NHMRC), and recommendations from most studies on acute pain management.

Despite the suboptimal pain treatment practices observed at the ED, the majority of patients expressed high levels of satisfaction with the pain management they received. These findings serve as a wake-up call to stakeholders in emergency healthcare(particularly in low- and middle-income countries (LMICs) such as Ghana)to intensify efforts in improving pain management services, with a specific focus on timeliness, to ensure more effective and efficient pain care.

The 184-minute delay represents a nearly four-fold deviation from even the extended 90-minute benchmark and a six-fold deviation from NHMRC guidelines. This finding necessitates urgent, systematic intervention at multiple levels of the emergency care delivery system

### Recommendations

Poor management of acute pain leads to chronic pain which is associated with high financial burden to the patient and health care system. Based on the findings from the present study, the following recommendations have been formulated:

1. Implementing nurse-initiated analgesia protocols for patients triaged with severe pain
2. Addressing structural barriers such as medication accessibility and ED workflow optimization
3. Regular auditing of DTA times with feedback to clinical staff
4. Emergency departments should implement systematic documentation of all analgesic interventions, including drug name, dose, route, and time of administration.
5. Regular audits should assess adherence to evidence-based guidelines such as the WHO analgesic ladder to ensure appropriate medication selection based on pain severity
6. Patient satisfaction surveys should be interpreted cautiously and not used as the sole metric of pain management quality.
7. Objective clinical outcomes, including pain score reduction and time to analgesia, must be prioritized alongside patient-reported satisfaction

## Supporting information

S1 File Acute pain data. (XLSX)

S2 File Questionnaire. (DOCX)
