# Supplementary material for: Assessment of acute pain and its management in an urban emergency department in Ghana
Source: PLoS One. 2026 Mar 20;21(3):e0343797. doi: 10.1371/journal.pone.0343797 (PMC13004330; doi:10.1371/journal.pone.0343797)
Supplement: S2 File — (DOCX) [file pone.0343797.s002.docx]

# Questionnaire

**An assessment of acute pain and its management in an urban Emergency Department in Ghana**

**DATA COLLECTION SHEET**

**Demographics**

Patient’s identification number…………………….

1) Gender (a) Male (b) Female

2) Age ………………………………….

3) Educational Status: (a) None (b) Primary (c) JHS (d) SHS e) Vocational (e) tertiary

4) Occupation …………………………….

5) **Triage Zone:** (a) Red (b) Orange (c) Yellow

6) Do you subscribe to NHIS? (a) Yes (b) No

**Pain characteristics**

7) Reasons for ED admission

Trauma a) Yes b) No

If Yes specify…………... If No specify ……………

8) Location of pain


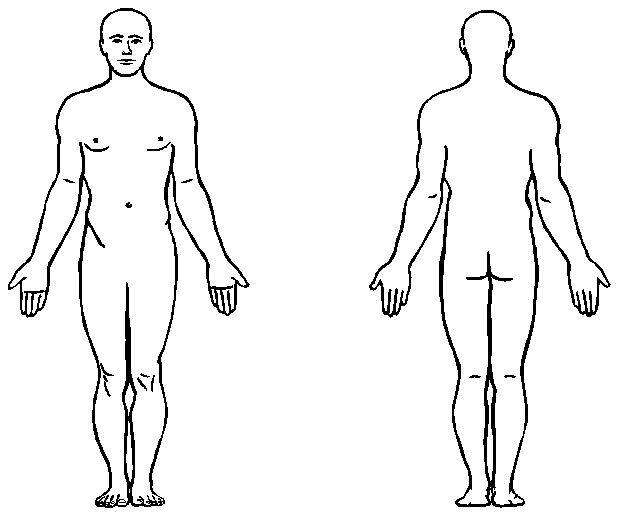


9) Duration of pain …………………………

**Pain score before analgesia**

10)

Grade the severity of pain?

|  | 0 | 1 | 2 | 3 | 4 | 5 | 6 | 7 | 8 | 9 | 10 |  |
| --- | --- | --- | --- | --- | --- | --- | --- | --- | --- | --- | --- | --- |
|  | No pain |  |  |  |  | Moderate | |  |  |  | Severe | |

**Waiting time**

11) Time of arrival at the ED ………………

12) Time seen by physician ……………….

13) Time of initial pain medication ………...

14) DTA Time ……………………………

**Medication**

15) Pain medication received……….

16) Route of administration of analgesic……………

**Pain score after analgesia**

17)

Grade the severity of pain now?

|  | 0 | 1 | 2 | 3 | 4 | 5 | 6 | 7 | 8 | 9 | 10 |  |
| --- | --- | --- | --- | --- | --- | --- | --- | --- | --- | --- | --- | --- |
|  | No pain |  |  |  |  | Moderate | |  |  |  | Severe | |

18) **Satisfaction**

(a) How satisfied were you with the overall treatment you received?

| 0 | 1 | 2 | 3 | 4 | 5 | 6 | 7 | 8 | 9 | 10 |
| --- | --- | --- | --- | --- | --- | --- | --- | --- | --- | --- |
| No satisfaction |  |  |  |  | Moderate | |  |  | Maximum satisfaction | |

(b) How satisfied were you with the staff warmth, respect, kindness, and willingness to listen?

| 0 | 1 | 2 | 3 | 4 | 5 | 6 | 7 | 8 | 9 | 10 |
| --- | --- | --- | --- | --- | --- | --- | --- | --- | --- | --- |
| No satisfaction |  |  |  |  | Moderate | |  |  | Maximum satisfaction | |

(c) How satisfied were you with the skills and competence of the staff?

| 0 | 1 | 2 | 3 | 4 | 5 | 6 | 7 | 8 | 9 | 10 |
| --- | --- | --- | --- | --- | --- | --- | --- | --- | --- | --- |
| No satisfaction |  |  |  |  | Moderate | |  |  | Maximum satisfaction | |

(d) How satisfied were you with the hours of treatment?

| 0 | 1 | 2 | 3 | 4 | 5 | 6 | 7 | 8 | 9 | 10 |
| --- | --- | --- | --- | --- | --- | --- | --- | --- | --- | --- |
| No satisfaction |  |  |  |  | Moderate | |  |  | Maximum satisfaction | |

(e) Would you recommend this treatment to someone you know who has an acute pain problem?

| 0 | 1 | 2 | 3 | 4 | 5 | 6 | 7 | 8 | 9 | 10 |
| --- | --- | --- | --- | --- | --- | --- | --- | --- | --- | --- |
| Not recommended |  |  |  |  | Moderately | |  |  | Strongly recommended | |
